# Application of sebomics for the analysis of residual skin surface components to detect potential biomarkers of type-1 diabetes mellitus

**DOI:** 10.1038/s41598-017-09014-6

**Published:** 2017-08-21

**Authors:** Satyajit S. Shetage, Matthew J. Traynor, Marc B. Brown, Thomas M. Galliford, Robert P. Chilcott

**Affiliations:** 1 Research Centre for Topical Drug Delivery and Toxicology, Department of Pharmacy, University of Hertfordshire, College Lane Campus, Hatfield, AL10 9AB UK; 2 MedPharm Ltd, 50 Occam Road, Surrey Research Park, Guildford, Surrey, GU2 7AB UK; 3 West Hertfordshire Hospitals NHS Trust, Watford General Hospital, Watford, WD18 0HP UK

## Abstract

Metabolic imbalance in chronic diseases such as type-1 diabetes may lead to detectable perturbations in the molecular composition of residual skin surface components (RSSC). This study compared the accumulation rate and the composition of RSSC in type-1 diabetic patients with those in matched controls in order to identify potential biomarkers of the disease. Samples of RSSC were collected from the foreheads of type-1 diabetic (n = 55) and non-diabetic (n = 58) volunteers. Samples were subsequently analysed to identify individual components (sebomic analysis). There was no significant difference in the rate of accumulation of RSSC between type-1 diabetics and controls. In terms of molecular composition, 171 RSSC components were common to both groups, 27 were more common in non-diabetics and 18 were more common in type-1 diabetic patients. Statistically significant (P* < *0.05) differences between diabetic and non-diabetic volunteers were observed in the recovered amounts of one diacylglyceride (m/z 594), six triacylglycerides (m/z 726–860) and six free fatty acids (m/z 271–345). These findings indicate that sebomic analysis can identify differences in the molecular composition of RSSC components between type-1 diabetic and non-diabetic individuals. Further work is required to determine the practical utility and identity of these potential biomarkers.

## Introduction

The human integument is coated with a thin layer comprising sebum, sweat, corneocyte debris and natural moisturising factors. Whilst generically referred to as sebum, the mixture is more accurately referred to as “residual skin surface components” or RSSC^1^.

Changes in the molecular composition of RSSC may arise as a result of local and/or systemic disease states^2–4^. Indeed, clinical conditions such as acne are associated with changes in both the secretion rate and the composition of sebum (Table 1). In addition, perturbations in the rate of sebum secretion have also been reported for hypothyroidism^5^, Turner syndrome^6^, Behçet’s syndrome^7^, Parkinson’s disease^8^ and rheumatoid arthritis^7^. Thus, the detection and quantification of disease-specific molecules present on the skin surface offer potential for the development of non-invasive diagnostic and prognostic techniques.

**Table 1:** Examples of clinical conditions that have been associated with changes in sebum secretion rate or sebum composition. Note that in this context, sebum is used as a generic term for residual skin surface components.

Condition	Observation	Reference(s)
Acne	Increased sebum secretion rates	51–54
	Decreased free fatty acid and increased triglyceride and wax ester content of sebum	52
	Decreased concentrations of linoleic acid in skin surface lipids proportional to an increased rate of sebum production	55, 56

Decreases in sebum secretion rates have previously been reported for diabetes mellitus^9, 10^. However, to date there are no reports of specific biochemical changes in RSSC associated with chronic disease conditions such as type-1 diabetes. Thus, the aim of this human volunteer study was to identify any differences in the secretion rate or composition of RSSC between type-1 diabetic and non-diabetic volunteers. The rationale for specifically considering type-1 diabetic patients in the current study was related to the lack of insulin observed in these individuals^11, 12^. Decreased insulin levels may affect lipid synthesis in the sebaceous glands, as insulin is considered to be an essential hormone for the normal growth and differentiation of sebaceous gland cells^13^. A successful outcome to this pilot study would provide proof of principle, allowing the design of further studies to investigate non-invasive biomarkers of type-1 diabetes present in RSSC that may have diagnostic or prognostic application. This study is novel in its approach to identifying diabetic patients based on skin lipid composition. Therefore, a broad method of analysis was required to allow for the detection of unknown lipid molecules and the identification of potential biomarkers (which are subject to future confirmatory studies).

## Results

### Rate of RSSC accumulation

The rate of RSSC accumulation did not differ significantly (P > 0.05, Mann–Whitney test) between type-1 diabetic (0.12 ± 0.06 mg cm^−2^, n = 55) and non-diabetic (0.13 ± 0.06 mg cm^−2^, n = 58) volunteers. There was no significant correlation (r^2^ = 0.004, P > 0.05, n = 51) between the amount of RSSC recovered and the clinically-confirmed duration of diabetes (data not shown). Blood concentrations of HbA1c in type-1 diabetic volunteers did not correlate (r^2^ = 0.001, P > 0.05, n = 54) significantly with the amount of RSSC recovered (data not shown).

### Composition of RSSC

**Figure 1:**
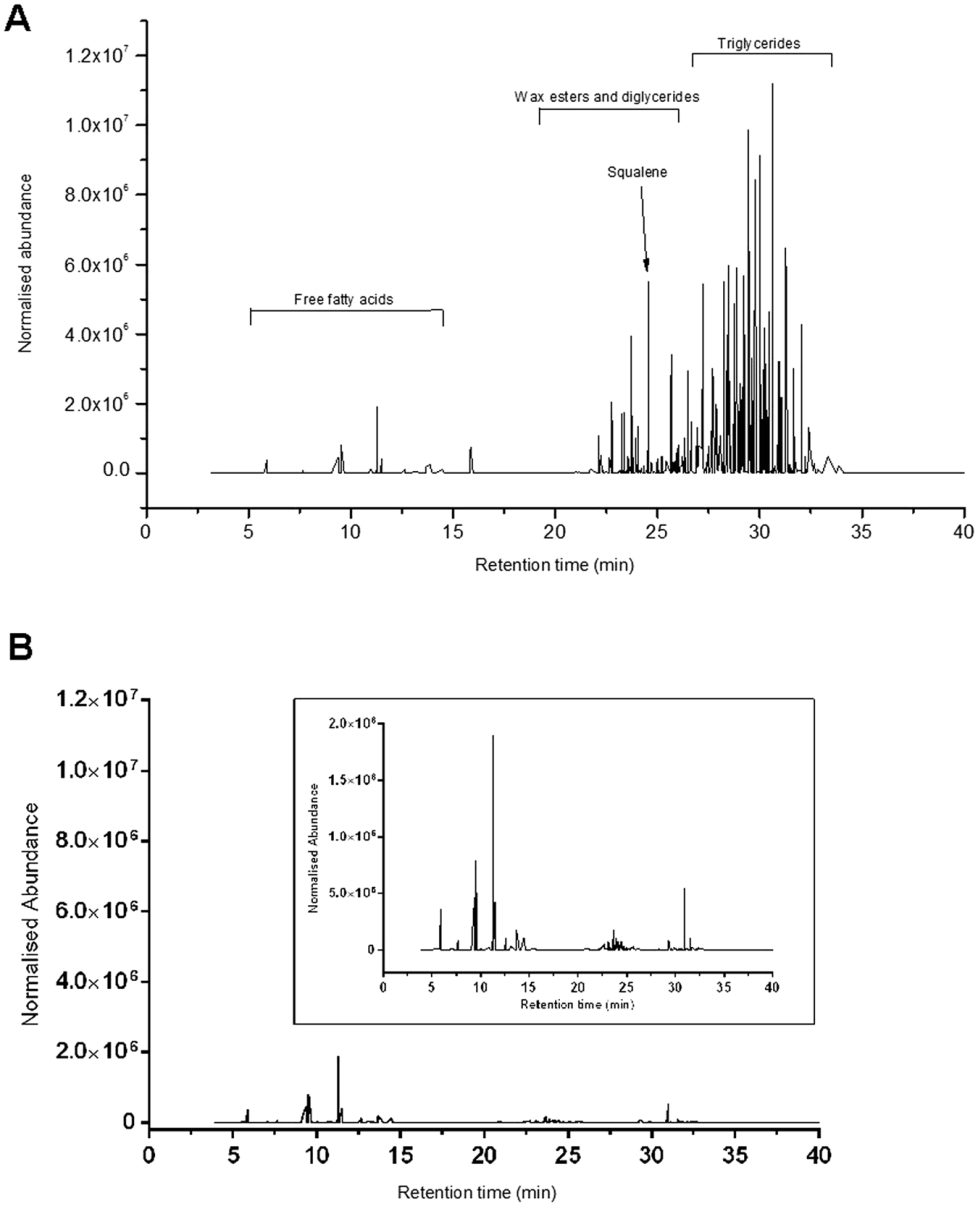
Representative chromatogram of human residual skin surface components. Distributions were reconstructed based on the normalised abundance of compound ions detected in both positive and negative ionisation analysis (**A**) and negative ionisation analysis only (**B**); showing inset zoom-in) by high performance liquid chromatography with atmospheric pressure chemical ionization mass spectrometry.

Analysis of RSSC by high-performance liquid chromatography (HPLC) with atmospheric pressure chemical ionization (APCI) mass spectrometry (MS) presented complex chromatograms indicating the presence of several RSSC components. A representative chromatogram constructed using the normalised abundance of compound ions is shown in Fig. 1.

**Figure 2:**
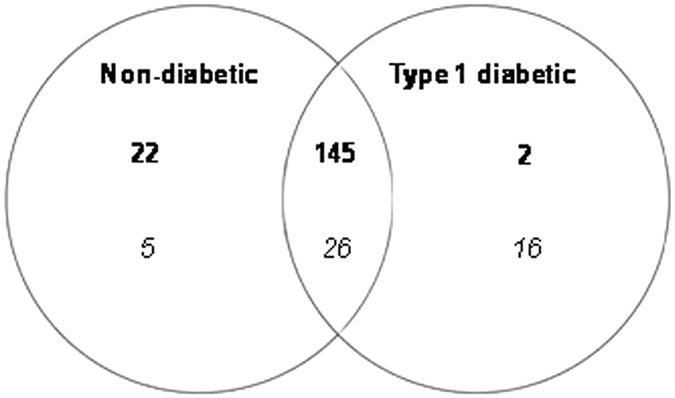
Commonalities and differences in the number of compound ions detected in positive ionisation (bold) and negative ionisation (italics) mode between type-1 diabetic (n = 44) and non-diabetic (n = 58) residual skin surface component samples.

In total, 216 compound ions were consistently identified from samples of RSSC recovered from type-1 diabetic and non-diabetic participants: the majority (78%) were detected in positive ion mode, with the remainder (22%) in negative ion mode (Fig. 2).

There were 171 compound ions common to both type-1 diabetic and non-diabetic RSSC. The majority (145) were detected in LC-MS positive ionisation mode, with 26 detected in negative ionisation mode (Fig. 2). The normalised abundance of 2 of the 145 common positive ions identified as triglycerides was more than twofold higher (P < 0.05) in non-diabetic volunteers (Table 2). No significant difference (P > 0.05) was observed in the normalised abundance of the 26 common negative ions.

**Table 2:** Comparison of ion distributions in diabetics and non-diabetics (positive ionisation). Compound ions with significantly different normalised abundance (mean ± standard error of the mean) in positive ion chromatograms of non-diabetic (n = 58) and type-1 diabetic (n = 44) volunteers. Neutral mass of the putatively identified compounds was calculated by subtracting NH_4_^+^ adduct ion (m/z 18.033). Two triglycerides (TG) were consistently present in both groups. One diglyceride (DG) and four other triglycerides were consistently present only in the non-diabetic group. The normalised abundance of these seven compound ions was more than twofold higher (P < 0.05) in non-diabetic volunteers than in type-1 diabetic volunteers.

Compound ion (m/z_RT)	Normalised Abundance Mean (±standard error of the mean)		Putative Compound Identification (Name and formula)
	Non diabetic (n = 58)	Type-1 diabetic (n = 44)	
**Not consistently present in Type-1 diabetic group**			
**594.52_30.04**	7.35 × 10^4^ (1.28 × 10^4^)	3.13 × 10^4^ (1.06 × 10^4^)	DG(15:0_18:3) C_36_H_64_O_5_
**726.59_27.60**	1.63 × 10^5^ (2.30 × 10^4^)	7.96 × 10^4^ (1.38 × 10^4^)	TG(13:0_13:0_15:0) C_44_H_84_O_6_
**797.67_28.57**	6.63 × 10^4^ (1.11 × 10^4^)	2.24 × 10^4^ (4.85 × 10^3^)	TG(12:0_16:0_18:0) C_49_H_94_O_6_
**844.71_29.28**	5.34 × 10^4^ (7.78 × 10^3^)	1.42 × 10^4^ (3.21 × 10^3^)	TG (16:0_17:2_17:2) C_53_H_94_O_6_
**860.71_30.72**	9.53 × 10^4^ (1.27 × 10^4^)	4.17 × 10^4^ (9.61 × 10^3^)	TG(17:1_17:1_17:1) C_54_H_98_O_6_
**Consistently present in both groups**			
**762.62_27.22**	1.75 × 10^5^(2.73 × 10^4^)	6.49 × 10^4^ (1.00 × 10^4^)	TG(12:0_12:0_20:3) C_47_H_84_O_6_
**776.62_27.64**	1.03 × 10^5^ (1.46 × 10^4^)	5.02 × 10^4^ (8.86 × 10^3^)	TG(15:1_15:1_15:1) C_48_H_86_O_6_

Twenty-two positive compound ions were consistently present only in the RSSC of non-diabetic volunteers (Fig. 2), of which five (1 diglyceride and 4 triglycerides) had a normalised abundance that was more than twofold higher (P < 0.05) than that of type-1 diabetic volunteers (Table 2). Two positive ion compounds were consistently present only in type-1 diabetic volunteers. However, the normalised abundance of these compound ions did not differ significantly between the two groups (P > 0.05). In negative ionisation mode, five compound ions were consistently present only in RSSC recovered from non-diabetic volunteers (Fig. 2). No significant difference (P > 0.05) was observed in the normalised abundance of these five negative ions. A further 16 compound ions were consistently present only in the type-1 diabetic volunteers; the normalised abundance of six of these compound ions (all free fatty acids) was more than twofold higher (P < 0.05) than in non-diabetic volunteers (Table 3).

**Table 3:** Comparison of ion distributions in diabetics and non-diabetics (negative ionisation). Compound ions with significantly different normalised abundance (mean ± standard error of the mean) in negative ion chromatograms of non-diabetic (n = 58) and type-1 diabetic (n = 44) volunteers. Neutral mass of the putatively identified compounds was calculated by addition of OAc^−^ adduct (m/z 59.01), except for compound 345.07_23.45, which had the mass of H_2_O-H adduct (m/z 19.02) applied. All six free fatty acids (FFA) were consistently present in type-1 diabetic volunteers and their normalised abundance was more than twofold higher (P < 0.05) in type-1 diabetic volunteers than in non-diabetic volunteers.

Compound ion (m/z_RT)	Normalised Abundance Mean ( ± standard error of the mean)		Putative Compound Identification (Name and formula)
	Non diabetic (n = 58)	Type-1 diabetic (n = 44)	
**271.11_23.45**	8.73 × 10^3^ (1.35 × 10^3^)	1.81 × 10^4^ (1.63 × 10^3^)	FFA C13:1n-5 (8-tridecenoic acid) C_13_H_24_O_2_
**273.12_23.45**	2.98 × 10^3^ (5.82 × 10^2^)	9.37 × 10^3^ (1.09 × 10^3^)	FFA C13:0 (Tridecylic acid) C_13_H_24_O_2_
**313.15_7.661**	2.72 × 10^4^ (6.00 × 10^3^)	7.42 × 10^4^ (1.67 × 10^4^)	FFA C16:1n-14 (Gaidic acid) C_16_H_30_O_2_
**315.11_10.10**	6.05 × 10^3^ (1.92 × 10^3^)	2.48 × 10^4^ (5.42 × 10^3^)	FFA C16:0 (Palmitic acid) C_16_H_32_O_2_
**341.14_11.24**	2.96 × 10^4^ (6.21 × 10^3^)	8.80 × 10^4^ (1.73 × 10^4^)	FFA C18:1n-9 (Oleic acid) C_18_H_34_O_2_
**345.07_23.45**	1.73 × 10^3^ (4.74 × 10^2^)	2.26 × 10^4^ (2.10 × 10^3^)	FFA C24:2n-15,19 (5,9-tetracosadienoic acid) C_24_H_44_O_2_

## Discussion

This study identified a number of significant differences in the composition of RSSC recovered from type-1 diabetic and non-diabetic volunteers. In particular, there were significant perturbations in the frequency and abundance of certain free fatty acids and (di and tri) glycerides. However, this study identified no diabetes-related effects in the rate of accumulation of RSSC. Moreover, the duration and severity (measured by HbA1c) of type-1 diabetes also showed no correlation with the rate of accumulation of RSSC. Thus, there appear to be subtle differences in the composition but not the quantity of RSSC secreted by type-1 diabetic and non-diabetic individuals.

The rationale for specifically considering type-1 diabetic patients in the current study was related to the lack of insulin observed in these individuals^11, 12^. Decreased insulin levels in these individuals may affect lipid synthesis in the sebaceous glands, as insulin is considered to be an essential hormone for the normal growth and differentiation of sebaceous gland cells^13^. A level of biomarker HbA1c ≥ 47.5 mmol mol^−1^ indicates the presence of diabetes^14, 15^. In clinically diagnosed diabetic patients, a level of HbA1c below 53.0 mmol mol^−1^ can be considered as good control of diabetes, whereas a level exceeding 53.0 mmol mol^−1^ is generally considered poor^16^. Non-physiological insulin profiles have been reported in patients with apparently well controlled diabetes^17^. Insulin is also known to affect the differentiation and proliferation of keratinocytes in rats^13, 18^ and it has been suggested that abnormal insulin signalling may also contribute to the dermal manifestations of diabetes^19^. In sebocytes, insulin exerts its effects through insulin-like growth factor-I receptors and upregulation of sterol regulatory element-binding proteins that, in turn, stimulate lipogenesis in sebocytes^20^. Insulin is also necessary for maximising the effect of growth hormone^13^. Given these multiple potential pathways through which abnormal insulin levels may affect sebum production, differences in sebum production between diabetic and non-diabetic individuals would appear to be a physiological inevitability. The evidence from the studies cited above would tend to predict a decrease in sebum secretion in response to low levels of insulin.

This study built on previous work that showed a decrease in the rate of sebum secretion in diabetic rats^21^ and humans^9, 21^. One previous study^9^ measured casual sebum levels using the Sebumeter™ technique; however, a limitation was the potential for sample-site contamination. In the present study, the subject’s forehead was cleaned before sample collection and protected for the duration of sample acquisition to ensure the measurement of RSSC accumulation was not unintentionally influenced by contamination of the measurement site, as per current recommendations and guidance^21–25^.

The results obtained in this study did not demonstrate any difference in the rate of accumulation of RSSC between type-1 diabetic and non-diabetic individuals, but did detect significant differences in composition. This is in agreement with a previous investigation that demonstrated no changes in the histological appearance of sebaceous glands, although a decrease in triglyceride synthesis following the onset of diabetes has been reported in mice^26^. The treatment with exogenous insulin in type-1 diabetic patients may be responsible for the non-significant difference in the rate of RSSC accumulation between the two groups (although according to the HbA1c data, diabetes was not well controlled in the majority of the individuals).

The biomarker HbA1c is a form of haemoglobin modified by the non-enzymatic attachment of glucose. As the lifespan of an erythrocyte is normally around 120 days^27^, HbA1c reflects average plasma glucose levels over an eight- to twelve-week period^28^. Sebocyte differentiation and sebum production normally takes 21–25 days^29^ and so the HbA1c marker provides a good indication of blood glucose over several sebum production cycles. In the current study, no correlation was observed between HbA1c level and the rate of RSSC accumulation. This result is in alignment with the observations of Sakai *et al*.^10^ in human subjects. The lack of correlation between HbA1c and RSSC recovery may be explained by the fact that HbA1c represents the overall accumulation of excess glucose and so does not take into account the dynamic nature of blood glucose concentrations, which may stimulate or inhibit the rate of sebum production.

Sebomic analysis using LC-MS indicated that several components of RSSC were common to type-1 diabetic and non-diabetic volunteers. However, the normalised abundance of two common triglycerides was higher in non-diabetic volunteers (Table 2). In addition to these components, one diglyceride and four triglycerides showed a higher prevalence (as well as an increased abundance) in non-diabetic volunteers. Such variability in the composition of RSSC may partly be attributable to the availability of substrates for lipogenesis pathways. Sebaceous glands use various substrates for lipid synthesis, including lactate, acetate, amino acids and glucose^30, 31^. Sebaceous glands also contain elevated concentrations of glycogen^30^. Studies using radioactive glucose with isolated sebaceous glands have demonstrated that glucose is incorporated into all sebaceous lipid classes^32^. However, the rate of incorporation of glucose is approximately half that of lactate and acetate^30^. Using lactate and acetate as precursors, the rate of squalene and wax ester synthesis is significantly higher than with glucose, whereas triglyceride synthesis is unaffected^30^. Lipids synthesised from glucose and lactate are very similar in composition^33^. Therefore, the effect of glucose on lipid synthesis could potentially alter RSSC composition, as postprandial hyperglycaemia and nocturnal hypoglycaemia are usually observed even in patients with well controlled type-1 diabetes^34, 35^. It is likely that diabetic patients use the same metabolic pathways of lipid synthesis as non-diabetics, but subtle differences in the availability of substrates for lipogenesis (caused by glucose imbalance) may introduce variability in the composition of sebum, which may explain the reduced abundance of diglycerides and triglycerides identified in the current study. Further work is required to investigate the effect of blood glucose concentrations on sebaceous gland activity in order to elucidate the mechanism through which glucose may influence the composition of skin surface lipids.

Interestingly, the abundance and prevalence of six fatty acids were greater in the diabetic group (Table 3), most probably as the result of increased lipase activity. Microbial lipase is responsible for the metabolism of triglycerides, resulting in the release of free fatty acids on the skin surface^36, 37^. A greater number of micro-organisms have been isolated from the skin surface of diabetic compared with non-diabetic individuals^38^. Enhanced lipase activity as a consequence of increased microbial density on the skin of diabetic patients may, therefore, be responsible for the higher abundance of fatty acids in diabetic volunteers observed in the current study. In contrast, non-diabetic volunteers would be expected to have a higher abundance of triglycerides on the skin surface, due to a correspondingly lower lipase activity: this was observed in the current study. A concurrent increase in diglyceride abundance (due to microbial hydrolysis of triglycerides) would also be expected in diabetic patients^39^. However, the only detectable change measured in this current study was a decrease in the normalised abundance of one diglyceride in diabetic patients (Table 2). This apparent anomaly cannot be explained without robust identification of this component and further work is required to identify alternative routes of diglyceride synthesis that might explain this result.

Whilst it is tempting to speculate that the statistically significant differences in RSSC composition measured in this present study may provide a basis for developing a non-invasive, diagnostic indicator of type-1 diabetes, further work is clearly required to (1) confirm the identity of the compound ions that differ between diabetic and non-diabetic individuals, and (2) identify the mechanism(s) underpinning the compositional differences between the diabetic and non-diabetic groups. Prior to instigating the study, no information existed regarding whether or which skin lipids were indicative of a diabetic condition; therefore, we were obliged to adopt a broad method of analysis. This effectively precluded the use of specific internal standards and external reference samples from which we could confirm the exact identity of the biomarkers putatively identified in this pilot study. Such pilot studies are important in identifying new areas of research that may have future impact. Thus, preliminary studies such as ours need to adopt an inherently flexible methodology to allow the detection of unknown outcomes, which would otherwise be precluded by adopting a hypothesis-driven approach that might limit detection to “known” lipid molecules. Sebomic analysis of RSSC may represent a promising strategy for identifying biomarkers of other chronic diseases that are known to be associated with changes in sebaceous gland biochemistry. For example, the presence of phosphorylated tau protein in the cytoplasm of sebaceous glands and altered sebum secretion rates have been reported in Alzheimer’s^40^ and Parkinson’s disease^8^.

In conclusion, a comparison of RSSC collected from type-1 diabetic and non-diabetic volunteers did not show differences in the rate of RSSC accumulation. However, significant differences in the composition of RSSC were noted between type-1 diabetic and non-diabetic individuals. The observed variability in the RSSC composition of diabetic volunteers may be indicative of an altered availability of substrates for lipogenesis. However, bacterial lipase activity is also likely to be a contributory factor in the decreased recovery of triglycerides and corresponding increase in free fatty acids observed in the RSSC of diabetic individuals. This study supports the feasibility of using RSSC as a non-invasive biomarker of type-1 diabetes.

## Methods

This study was conducted in accordance with the principles of the Declaration of Helsinki and was independently approved by the NHS South Central–Hampshire A Research Ethics Committee.

### Volunteers

Ethical approval to collect RSSC from non-diabetic volunteers was granted by the School of Pharmacy and Postgraduate Medicine Ethics Committee with Delegated Authority, University of Hertfordshire, Hatfield, UK (ethics approval number: PHAEC/10-25). For the collection of RSSC from type-1 diabetic patients, ethical approval was obtained from the National Research Ethics Service (NRES) Committee London-Bloomsbury (ethics approval number: 13/LO/1196). Collection of RSSC from non-diabetic volunteers was performed at the University of Hertfordshire (UK) while patients with clinically diagnosed type-1 diabetes were recruited at Watford General Hospital (UK) and Hemel Hempstead Hospital (UK). All volunteers participating in the study provided written informed consent. Demographic data were obtained using a questionnaire. Clinical information (e.g. duration of illness, glycated haemoglobin [HbA1c] blood concentrations, current medication, etc.) was obtained from each type-1 diabetic patient’s hospital record. Patients in the type-1 diabetic group remained on exogenous insulin treatment throughout the experiment. Type-1 diabetic and non-diabetic groups were matched as far as possible for age and sex. Previous studies^1, 41^ have indicated that the rate of RSSC production is not affected by an individual’s ethnicity. Table 4 summarises the self-reported demographics of all participants.

**Table 4:** Summary of residual skin surface component analysis, performed using gravimetry and liquid chromatography with atmospheric pressure chemical ionization mass spectrometry, in relation to volunteer details.

Analysis	Total (n)	Sex (n)	Age	Ethnicity (n)	
			(years)*	White	Asian
**Gravimetry**	Type-1 diabetic (55)	Male (31)	39 ± 15	26	5
		Female (24)	35 ± 14	23	1
	Non-diabetic (58)	Male (25)	39 ± 15	19	6
		Female (33)	38 ± 15	29	4
**LC-APCI-MS**	Type-1 diabetic (44)	Male (25)	36 ± 15	21	4
		Female (19)	32 ± 13	18	1
	Non-diabetic (58)	Male (25)	39 ± 15	19	6
		Female (33)	38 ± 15	29	4

*Data given as mean ± standard deviation.

### Collection of RSSC

Collection of RSSC from volunteers was performed using a previously validated cigarette paper method^1, 42^ over a four-month period (February-June, 2014). Briefly, volunteers were allowed to acclimatise to the study room conditions (18–26 °C, 50–60% relative humidity) for at least 15 minutes before their forehead was cleaned with an isopropyl alcohol-based wipe (Fastaid™, Robinsons Healthcare, Worksop, UK) prior to the application of four pieces of dry, pre-weighed cigarette paper (previously washed with diethyl ether). Each paper was covered with a piece of aluminium foil which was fixed to the forehead using Micropore™ (3 M UK Plc., Bracknell, UK) adhesive tape. This assembly was held in place with an elastic head band. After removal from the forehead, the cigarette papers were dehydrated (by passive evaporative loss) in plastic sample cups covered with pierced Parafilm™ in a fume cupboard for a minimum period of two hours prior to storage at ambient temperature.

### Analysis of RSSC

The rate of RSSC production was determined by gravimetric analysis performed using a 10-µg resolution fine balance (Mettler Toledo AX205; Mettler-Toledo Ltd., Leicester, UK) placed on a vibration-proof table. The balance was encased in a weighing cabinet (Bigneat Ltd., Hampshire, UK) to reduce draught artefacts. Each piece of cigarette paper was weighed before and after RSSC collection, the difference in weight being ascribed to RSSC recovered from the skin surface.

To identify compositional differences, RSSC present on the cigarette papers was extracted using hexane. The solvated RSSC samples were then dried by purging with nitrogen gas until all the hexane was visibly removed. The dried sample was then resuspended in 1 mL chloroform:methanol (2:1) using a roller mixer (Stirling mixer, Sandrest Ltd., Sussex, UK) for a minimum period of two hours. The sample was then transferred to a clean vial for LC-APCI-MS analysis. Chemical analysis was performed using a ThermoScientific™ Ultimate 3000 HPLC system, comprising an RS 3000 quaternary pump, Ultimate 3000 RS auto-sampler and a column oven, connected to a ThermoScientific™ MSQ™ single quadrupole mass spectrometer (Thermo Fisher Scientific, Hemel Hempstead, UK). A reverse phase Zorbax SB C8 column (internal diameter 2.1 mm, length 150 mm, particle size 1.8 µm) was purchased from Agilent Technologies (Berkshire, UK) and used for chromatographic separation. The column was maintained at a temperature of 60 °C. The mobile phase was drawn from reservoirs containing 95:5 methanol:isopropyl alcohol (A) and 10 mM aqueous ammonium acetate (B). A gradient program was employed to optimise chromatographic separation: 0–1 min: 70% A; 20 min: 99% A; 45 min: 99% A; 55 min: 70% A; 60 min 70% A. The mobile phase flow rate was maintained at 0.25 mL min^−1^ throughout the 60-min run time. The APCI technique was used for ion generation (corona discharge 3 kV, probe temperature 400 °C, cone voltage 50 V). Ions were scanned in the range of 100 to 1000 Da to obtain a total ion scan in both positive and negative ionisation modes. ThermoScientific™ Chromeleon (version 3.8) software was used for the instrument control, data collection and initial data processing.

The data acquired for each sample were subsequently converted to “raw” file format and processed using Progenesis QI software (Nonlinear Dynamics, Newcastle Upon Tyne, UK). Spectral data alignment, peak detection (“peak picking”), background subtraction, normalisation and comparison of normalised abundance was performed using automated algorithms provided by the Progenesis QI software. Peak detection was performed on the intensity of compound ions, excluding events with a chromatographic (peak) width of less than 0.1 min. The abundance of compound ions in each sample was normalised against the abundance of compound ions in a reference chromatogram. An automated algorithm within the Progenesis QI software was used to select the most representative chromatogram among all the samples for use as a reference chromatogram. The final list of compound ion abundances was transformed using an inverse hyperbolic (arcsinh) function. The transformed data were then used to calculate any significant differences in the normalised abundance between groups. Such an “omics” approach is widely used in fields like metabolomics and proteomics for the processing of extremely complex biological data to enable a system level understanding of molecular interactions and dependencies in biological systems^43–45^. The prevalence of compound ions in each volunteer group was calculated as a percentage. A compound ion which was present in more than 75% of the population of a group was considered as a characteristic feature of that group (referred to as a “consistent” compound ion). The 75% threshold was adopted from previous (proteomic and metabolomics) biomarker studies^46–48^. The normalised abundance of consistent compound ions was then compared between the type-1 diabetic and non-diabetic volunteers. Using the mass-to-charge ratio (m/z), the components of RSSC were putatively identified based on literature reports^49^ and lipid databases^50^, namely the LIPID Metabolites and Pathways Strategy (LIPID MAPS®) databases.

### Statistical analysis

A commercially available software package (Statistical Package for the Social Sciences; SPSS Inc., Chicago, IL, USA, version 20) was used to perform the statistical analysis. The Kolmogorov–Smirnov test was used to determine the normality of the dataset and groups were compared by Mann–Whitney test. The transformed normalised abundance of compound ions detected in LC-APCI-MS analysis was compared by t-test using Progenesis QI software. A P-value < 0.05 was considered to be significant.
